# Expanding the Therapeutic Window for CAR T Cell Therapy in Solid Tumors: The Knowns and Unknowns of CAR T Cell Biology

**DOI:** 10.3389/fimmu.2018.02486

**Published:** 2018-10-26

**Authors:** Keisuke Watanabe, Shunichiro Kuramitsu, Avery D. Posey, Carl H. June

**Affiliations:** ^1^Center for Cellular Immunotherapies, Perelman School of Medicine at the University of Pennsylvania, Philadelphia, PA, United States; ^2^Parker Institute for Cancer Immunotherapy, University of Pennsylvania, Philadelphia, PA, United States; ^3^Corporal Michael J. Crescenz VA Medical Center, Philadelphia, PA, United States; ^4^Department of Pathology and Laboratory Medicine, Perelman School of Medicine at the University of Pennsylvania, Philadelphia, PA, United States

**Keywords:** T cell biology, chimeric antigen receptors, immune synapse formation, immunotherapy, cancer immunology

## Abstract

A major obstacle for chimeric antigen receptor (CAR) T cell therapy in solid tumors is the lack of truly tumor-specific target antigens, which translates to the targeting of tumor-associated antigens (TAAs) overexpressed on tumors but shared with normal organs, raising safety concerns. In addition, expression of TAAs in solid tumors is particularly heterogeneous. In this regard, it is critical to deeply understand the sensitivity of CAR T cells, especially against low-density targets and the possible therapeutic window of antigen density targeted by CAR T cells. In this review, we discuss the recent findings of mechanisms of antigen recognition through CAR, including immunological synapse formation, and the impact of target antigen density for induction of distinct T cell functions. We also discuss rational strategies to adjust and expand the therapeutic window for effective and safe targeting of solid tumors by CAR T cell platforms.

## Introduction

Chimeric antigen receptor T cell (CAR T cell) therapy has shown significant efficacy in hematological malignancies (1–3). Recently the U.S. FDA approved two types of CD19-targeting CAR T cells, tisagenlecleucel (Kymriah™–Novartis) in leukemia (August 2017) and lymphoma (May 2018) and axicabtagene ciloleucel (Yescarta™–Kite) in lymphoma (October 2017). The compelling success of CD19-specific CAR T cell therapies propels the development of CARs that can induce similar efficacy in solid tumors; however, the process is faced with multiple challenges that must be addressed to achieve sufficient efficacy.

Among the many challenges of CAR T cell therapy in solid tumors, a major obstacle is the lack of truly tumor-specific target antigens, which forces cellular immunologists to target tumor-associated antigens (TAAs) overexpressed on tumors but also expressed on normal tissues and organs, raising safety concerns. For instance, fatal cytokine release syndrome (CRS) has been reported from the targeting of human epidermal growth factor receptor 2 (HER2) with CAR T cells due to the recognition of low-levels of HER2 expressed on the normal cells of lung epithelium (4). Also, carbonic anhydrase IX-specific CAR T cells in renal cell cancer induced liver toxicities (5) and carcinoembryonic antigen (CEA)-specific transgenic T cell receptor (TCR) T cells induced severe colitis in colon cancer patients (6). In addition, the tumor microenvironment (TME) of solid tumors is particularly immunosuppressive, which prevents effective anti-tumor immune responses. The immunosuppressive TME contains multiple components including physical barriers, such as a dense extracellular matrix; dysfunctional epithelial cells; metabolic checkpoints, such as hypoxia and immunological barriers, such as immunosuppressive cytokines/molecules and immunosuppressive immune cells. To target such tumors effectively, multiple factors impacting efficacy and toxicity must be simultaneously addressed.

In this regard, it is critical to deeply understand CAR T cell biology and multiple factors that can affect the therapeutic window of CAR T cell therapies. In this review, we discuss the recent findings of mechanisms of antigen recognition through CARs, including immunological synapse (IS) formation, impact of target antigen density for induction of distinct T cell functions, and the kinetics of target cell killing. We also discuss rational strategies to adjust and expand the therapeutic window for effective and safe targeting of solid tumors by CAR T cell platforms.

## Basics of CAR T cell biology

While basic mechanisms by which T cells interact with targets through T cell receptors have been intensively investigated, those of CAR-target interactions are less well understood. As CARs consist of combined parts of the TCR complex and antibodies, it will be valuable to discuss the similarities of CARs to endogenous, unmodified TCR T cells and define distinct differences of CARs to better understanding CAR T cell biology (Table 1).

**Table 1:** Comparison of CAR and TCR T cell biological factors.

**FACTORS**	**TCR**	**CAR**
Components	Heterodimer	Single chain (Dimerized)
Costimulation (e.g., CD28, 4-1BB pathways)	Separated (in trans)	Linked (in cis; 2nd and 3rd generation CAR)
Coreceptor involvement	Yes (CD4, CD8, and CD45)	Yes (CD45, unknown for CD4, and CD8)
Target	MHC/peptide complex	Surface antigen^*^
Typical affinity of receptor	Lower (K_d_:10^−4^ M to 10^−6^ M)	Higher (K_d_:10^−6^ M to 10^−9^ M)
Required number of Ag to recognize	One	100 or less^**^
Hierarchical threshold antigen density for T cell functions	Yes	Yes
Immune synapse formation	Yes (Systematic “bull's eye” structure)	Yes (Disorganized)
Time required to form stable and functional immune synapse	Longer (5–10 min.)	Shorter (<2 min.)
Serial killing	Yes	Yes

* *Some of CARs have been developed to recognize MHC/peptide complex*.

** *Not tested precisely for target with under 100 target molecules*.

The TCR is a heterodimer of two subunits: a TCRα subunit and a TCRβ subunit. Each subunit contains a variable region domain (V) and a constant region domain (C), which is followed by a transmembrane region. Each V domain contains three complementarity-determining regions (CDRs), which interact with peptide presented on the major histocompatibility complex (MHC). The TCR itself does not possess signaling domains, requiring intracellular signaling to be initiated by the CD3 complex. The CD3 complex consists of three dimers, CD3ζε and CD3δε heterodimers and CD3 ζζ homodimer (7). The CD3γ/δ/ε subunits each consist of a single extracellular immunoglobulin (Ig) domain and an immunoreceptor tyrosine-based activation motif (ITAM), whereas CD3ζ has a short extracellular domain (ECD) and three ITAMs (8, 9). TCR and CD3 subunits form a complex on the T cell surface (TCR-CD3 complex).

CARs are synthetic chimeric proteins that are introduced into T cells to redirect antigenic specificity and enhance cellular functionality (10). CARs typically consist of a single-chain variable fragment (scFv) from a mAb, an extracellular spacer region (termed hinge), a transmembrane domain, CD3ζ signaling domain, and usually one or two costimulatory domain(s) for second-generation or third-generation CARs, respectively (11–14). Atypical constructions of CARs utilize receptor ligands or peptides as the extracellular antigen-recognition domain, such as zetakine CARs—e.g., interleukin-13 receptor alpha 2 (IL13Rα2) zetakine CARs (15). CARs endow T cells with the benefit of directly binding surface antigens via scFv (antibody recognition) in an MHC-independent manner, which allows activity from the same CAR molecule in both CD4 and CD8 T cells and reactivity against patient tumors regardless of histocompatibility. CARs can transmit signals through CD3ζ and costimulatory domains simultaneously to the T cell, which can induce a stoichiometric and potentially ideal activation of T cells.

## How do CARs trigger immunological synapse formation and transmit signaling?

**Figure 1:**
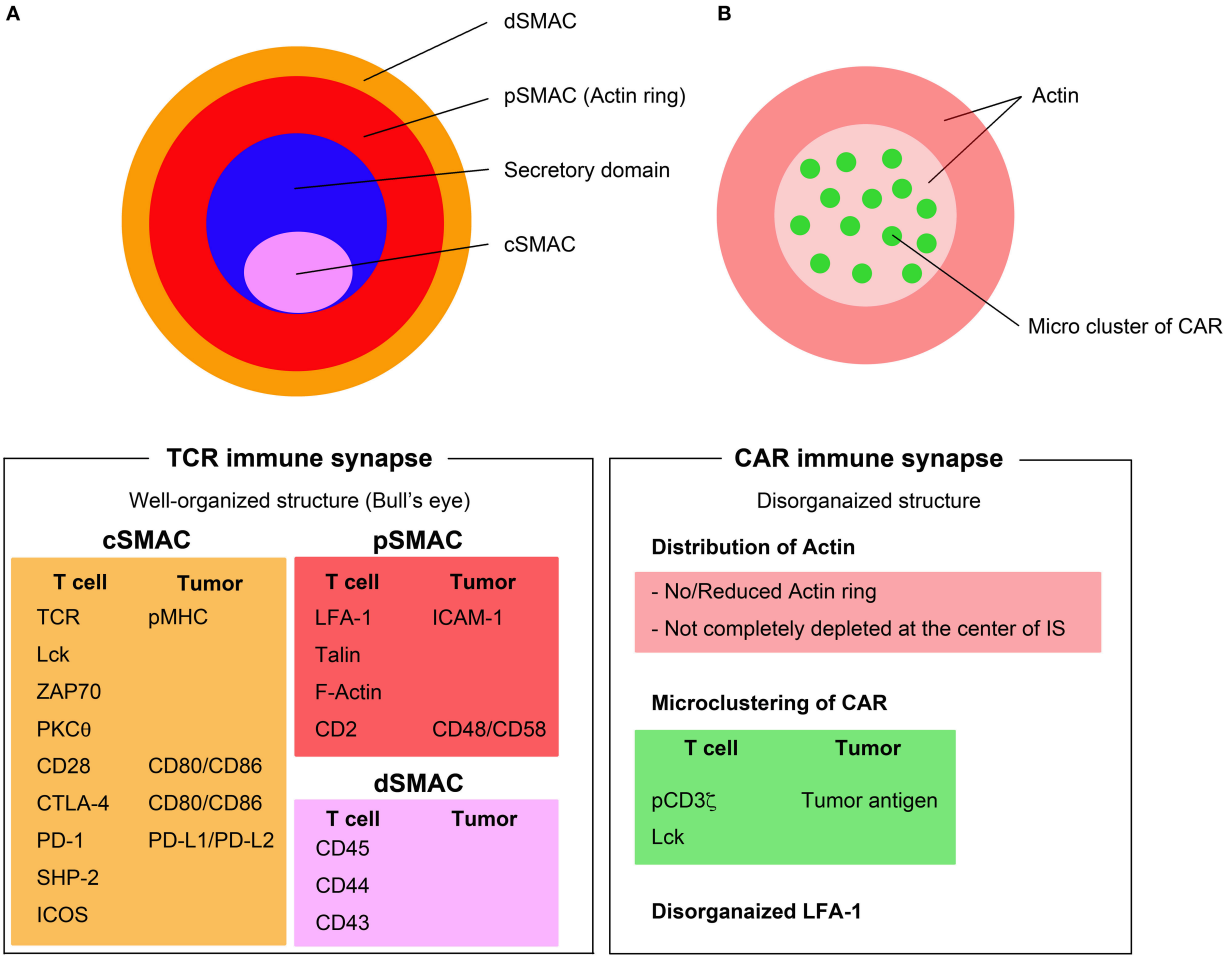
Immunological synapse formation through TCRs and CARs. **(A)** TCR immune synapse shows a well-organized bull's eye structure including the central supramolecular activation complex (cSMAC) (pink), the peripheral SMAC (pSMAC) (red), and distal SMAC (dSMAC) (orange). **(B)** CAR immune synapse (right) displays disorganized structure with no/reduced actin ring and microclusters of CAR/tumor antigen in a disorganized pattern. Major components of the TCR and CAR immune synapse are listed below the figures.

T cell activation is mediated through highly organized and dynamic interaction of TCRs with MHC-peptide complexes, referred to as an IS. A matured IS is an aggregation of TCR-based signalosomes that induce T cell responses. The IS is defined by three concentric rings of clustered molecules (Figure 1A). The inner circle of an IS is termed the central supramolecular activation cluster (cSMAC), where TCR signaling takes place. The cSMAC contains most of the TCR-MHC-peptide complexes, CD28, PKC-θ, and Lck, whereas peripheral SMAC (pSMAC) contains proteins involved in cell adhesion, such as integrin LFA-1, cytoskeletal linker talin, and ICAM1. Large molecules, such as CD43 and CD45, are excluded from the pSMAC and make up the distal SMAC (dSMAC). Inhibitory and costimulatory molecules, such as PD-1, CTLA-4, and ICOS also are aggregated at the region of IS and play crucial roles in the regulation of T cell activation (16).

Secretion of lytic granules occurs within a variant of IS (namely secretory synapse) between cytotoxic T lymphocytes (CTLs) and target cells. The secretary synapse has two separate and distinct domains in cSMAC: one is a signaling domain, which contains the signaling proteins, and another is a secretory domain for exocytosis of cytokines, perforins, and granzymes. Stinchcombe et al. demonstrated that the transient polarization and docking of the centrosome to the plasma membrane, which is controlled by Lck signaling, has an important role in the mechanism of directing this secretion (17–19).

The intracellular signaling downstream of CARs and the mechanisms of the IS formed by CARs have not been extensively studied. It has been demonstrated that CAR clustering, ZAP70 recruitment to IS, and exclusion of CD45 outside of IS occurs between CD19-specific CAR T cells and target cells that is similar to TCR activation. Downstream signaling molecules of the TCR, such as CD3ζ, LAT, Lck, and ZAP70 are phosphorylated after CD19-CAR T cell activation by autologous CD19^+^ B cells (20). In this study, third-generation CAR T cells had a significantly higher phosphorylation status on downstream TCR signaling molecules, and another study demonstrated that third-generation CARs, specifically those incorporating CD28 and 4-1BB costimulatory domains, induced a stronger PI3K/Akt activation when compared to second-generation CAR T cells upon *in vitro* exposure to antigen (21).

The formation of CAR IS has characteristics unlike the structure of TCR IS. The CAR IS does not present a systematic bull's eye structure, which is a characteristic feature of TCR IS. Organization of the actin ring in CAR IS is poor and actin may not be not completely diminished at the center of CAR IS (22). LFA-1 is disorganized and CAR-tumor antigen complexes form microclusters that are randomly distributed at the CAR IS (23) (Figure 1B). While TCR IS requires 5–10 min to form the bull's eye structure, the CAR IS might not need to form these stable structures because the disorganized multifocal pattern of CAR IS is sufficient to rapidly induce significant proximal signaling, which occurs within a short period of time (<2 min). Another important part of IS biology is the delivery of cytotoxic granules, including perforin and granzymes, to the IS mediated by microtubule organizing center (MTOC) (24). The rapid but short duration of proximal signaling of CAR IS also induces rapid MTOC migration to the IS and accelerates the delivery of granules (23). Although the mechanisms of CAR IS have gradually been revealed, it is still unclear whether the differences in CAR IS structure correlate with the efficacy of CAR T cells.

Soluble forms of CAR ligands, such as CD30, mesothelin, and CEA, that exist in monomeric forms cannot trigger CAR signaling (25–27), which is reasonable since they will not induce CAR dimerization. However, CAR T cells can potentially recognize soluble ligands that can exist in oligomeric forms, such as TGF-β, even without cell-cell interaction. Chang et al. recently demonstrated that TGF-β captured by an anti-TGF-β CAR could induce an IS, mimic actin-dependent CAR dimerization, and trigger T cell signaling (28). They also showed that the CAR response to the soluble ligands can be tuned by adjusting the extracellular spacers and the intracellular signaling domains of CARs. These findings reveal mechanisms by which the structures of CARs influence signaling and can also lead to strategies of engineering CAR T cells to overcome tumor immunosuppression by converting TGF-β from a potent immunosuppressive cytokine to a CAR T cell activator.

## What is the target density threshold for CAR T cell recognition?

It has been demonstrated through fluorescence microscopy that, under optimal conditions, as few as one peptide-MHC complex is sufficient to trigger T-cell activation, IL-2, and TNF-α secretion (29, 30), while a contradictory report suggested that four peptide-MHC complexes are the minimum required amount of agonists for half-maximal activation and calcium flux of CD4^+^ T cells (31). This high sensitivity of TCR signaling may reflect the unique role of the TCR, which requires the detection of a very rare foreign peptide presented on MHC in the presence of thousands of presented self-peptides. Orchestrated assembly of the receptor complex system may provide such high sensitivity while retaining specificity. The co-receptors CD4 and CD8 also participate in the binding and proximal signaling upon TCR interaction with peptide-MHC. For instance, CD4 acts to reduce the amount of peptide-MHC required from over 30 molecules/target cells to just one molecule (29). Interestingly, TCRs have a hierarchical threshold of antigen density for induction of cell lysis, proliferation, and cytokine production (32), where less antigen density is required for cell lysis than for cytokine production. This phenomenon is observed in the single cell levels but not as a T cell population (33).

To address the question of thresholds for CAR activation, Watanabe et al. investigated the density of CD20 required to activate CD20-specific CAR T cells (CD28 co-stimulation domain) with target cells expressing ~200–250,000 CD20 molecules per cell (34). Target cells expressing the lowest density of CD20 within the set of the target cells (~200 molecules/cell) could induce lysis by CAR T cells. This data was consistent with a previous report that CAR targeting a tumor-specific glycoepitope of murine OTS8 that could lyse target cells with similarly low density (~200 molecules/cell) of target antigen (35). This study also demonstrated that the CAR format is more sensitive than bi-specific T cell engagers (BiTEs) constructed with the same scFv.

Watanabe et al. also demonstrated that the target antigen density that is required to induce T cell proliferation and cytokine production was higher than that required to induce CAR mediated lysis: CD20-specific CAR T cells could lyse target cells with 200 molecules/cell, but cytokine production and T cell proliferation required a higher density of CD20, nearly 5,000 molecules/cell. In addition, recently Walker et al. investigated target antigen density required to activate ALK-specific CAR T cells with the 4-1BB co-stimulatory domain using Nalm6 cells expressing various densities of ALK (~18,000–450,000 molecules/cell) (36). In this model, production of IFN-γ, IL-2, and TNF-α showed a sharp threshold dependence on tumor antigen density and there was a significantly higher threshold for IL-2 production compared to IFN-γ production. IL-2 production required 60,000 molecules/cell and IFN-γ production required 30,000 molecules/cell to induce a half maximal response. CAR T cells could lyse target cells with the lowest ALK expression in this target cell panel (~18,000 molecules/cell); however, another panel of target cells with much lower ALK expression will be required to determine the absolute minimal density required for lysis. Liu et al. demonstrated that affinity-tuned anti-HER2 CARs consisting of scFvs derived from high affinity antibodies could degranulate when targeting very low HER2-expressing cells, where the expression was below detection capabilities by flow cytometric analysis (37). Although the number of target molecules was not determined in this study, this result suggests that CARs have a considerably lower threshold of antigen expression for target cell lysis; this threshold may be CAR- or scFv-dependent. There is a one-log difference in the threshold of IFN-γ production between the reports of Watanabe et al. and Walker et al. a discrepancy that might result from differences in the CAR constructs (e.g., affinity of scFv, hinge, co-stimulatory domain) or from the density of CAR expression, two features that could be utilized to more precisely enable control of CAR T cell activation.

These results suggest that CAR T cells can recognize target cells with considerably low levels of target antigen and that they have hierarchical T cell signaling thresholds for cell lysis, proliferation and individual cytokine production. It is likely that each T cell subset or each single T cell has a distinct threshold to be activated, as shown in TCR T cells, but this has not yet been investigated for CAR T cells.

## Do CAR T cells work as serial killers?

Endogenous T cells and NK cells can sequentially lyse multiple target cells (serial killing), which is likely to be necessary for tumor eradication (38, 39); however, the ability of CAR T cell to mediate serial killing and the kinetics of target cell lysis had not been fully demonstrated until recently. Davenport and colleagues tested the functions of cytotoxic T lymphocytes (CTLs) activated through either the endogenous TCR or an ectopically expressed CAR using a novel transgenic mouse model in which individual T cells co-expressed two antigen receptors (OT-I TCR and anti-HER2 CAR) (40). The authors clearly demonstrated that CAR T cells were serial killers using time-lapse video microscopy; approximately 22% of CAR T cells sequentially delivered a lethal hit to two or three tumor cells and the frequency of serial killing through the CAR was comparable to that through the TCR. Using kinetic analysis of tumor cell killing via a real-time impedance-based assay, the authors showed that CTLs elicited equivalent killing kinetics of target cells regardless of whether recognition occurred through the TCR or CAR for the first 20 h, but CAR-mediated lysis slowed its cytolytic kinetics compared to TCR-mediated lysis after 20 h. This difference in sustained lysis kinetics may be explained by CAR downmodulation after stimulation upon the antigen recognition, which can be ameliorated through TCR-based expression of CAR (41).

## How does CAR affinity affect T cell functions?

T cell activation is regulated by the interaction between the TCR and MHC-peptide complex and the major factors that have influence on the sensitivity of activation are target antigen density and TCR affinity. One of the major immunosuppressive mechanisms in the cancer microenvironment is failed antigen recognition due to low-affinity TCR and cancer associated peptide-MHC complex interactions (42, 43). TCR affinities to self-derived peptides, such as cancer antigens, are lower than TCR affinities to pathogen-derived antigens (44). Therefore, it is generally more difficult to isolate T cells that have sufficient sensitivity to TAAs than to identify pathogen-derived antigen-specific T cells from patients, and this is the first hurdle of adoptive cell therapies (ACTs) (45).

High TCR affinity is, on the other hand, accompanied by autoimmune responses, which sometimes leads to serious adverse events when patients are treated with ACTs. Although the affinity of TCRs is known to be μM range (K_d_:10^−4^M−10^−6^M), Zhong et al. reported that T cell antitumor activity and autoimmunity are closely coupled but plateau at a defined TCR affinity of 5–10 μM, which suggests that ACT utilizing supra-physiologic, high-affinity TCRs does not improve efficacy (46, 47).

It has also been reported that a small number of peptide-MHC complexes can achieve a high TCR occupancy since a single complex can serially engage and trigger hundreds of TCRs (48–50). Altogether, this suggests a model where the ideal affinity of TCR should provide interaction sufficiently long enough to transduce proximal signaling but appropriately short to detach and allow as many TCRs to encounter MHC-peptide complexes as possible.

The influence of the scFv affinity on CAR T cell functional response is still incompletely understood. In general, CARs constructed with scFvs possess higher affinity (in the nM range, K_d_:10^−6^ M−10^−9^ M) compared to native TCR affinities. Since most TAAs are highly expressed on tumors and at lower levels on normal tissues, it is essential to consider the threshold of the stimulation to yield optimal specificity of CAR-redirected T cell activation since there is a risk that increasing the affinity of CARs will lead to serious adverse effects due to on-target, off-tumor recognition (37, 51). The high affinity of the 4D5 (trastuzumab) scFv may be responsible for the fatal pulmonary toxicity and CRS that was attributed to anti-HER2 CAR reactivity against low level HER2 expression in the normal lung (4). As mentioned before, one strategy to increase the therapeutic index for TAA such as HER2 is affinity tuning of the scFv to generate HER2-specific CAR T cells unable to degranulate in response to normal human primary cells with low level HER2 expression (37).

Similar to native T cells, CAR T cells can also kill multiple target cells in a sequential fashion. However, tumor cells can be eliminated more rapidly when stimulated through CARs than through TCRs because CARs can dissociate from dying tumor cells more rapidly than TCRs (40). Hence, increasing the affinity of CAR T cells may reduce or prevent serial killing, promote T cell exhaustion, and decrease the generation and persistence of central memory and effector phenotype T cells (52), or increase the loss of T cells through activation-induced cell death (53).

## Selection of target antigens for solid tumors

A critical part of adoptive T cell therapy is the selection of the target antigen, in order to deliver sufficient efficacy and minimize toxicity. Some CARs targeting tumor-specific antigens have been developed pre-clinically, including CARs targeting aberrantly glycosylated oncogenes, such as the Tn glycoform of MUC1 (54), and tumor-specific activating forms of integrin (55), and clinically, such as CARs targeting the tumor-specific transcriptional variants EGFRvIII in glioblastoma (56). In the absence of more cancer-specific targets, CAR T cell therapies will most likely continue to target TAAs for solid tumors that also exhibit expression on normal tissues. Indeed, most of ongoing clinical trials of CAR T cell therapies for solid tumors are targeting such TAAs (57).

It is critical to know whether normal tissues express the antigen and its expression levels in order to predict potential toxicities. Several public databases of antigen expression on the normal tissues are available based on gene expression (RNAseq or microarrays) or immunohistochemistry (IHC). However, such technologies contain limitations and pitfalls. For gene expression analysis, antigens expressed by very rare but critical cells may be underestimated. In addition, it may not be possible to distinguish if expressed genes are derived from tissues or from infiltrating cells. For instance, some databases suggest that human intestine is CD4 positive; however, this expression likely represents infiltrated CD4 T cells rather than intestinal tissues themselves. The accuracy of IHC staining in public databases largely depends on the quality of the antibody, its affinity and the epitope for the antigen. For instance, the cancer-specific Tn glycoform of MUC1 recognized by the high-affinity antibody developed by (58) is extremely rare and unlikely to be identified in public IHC databases. Instead, researchers are more likely discover staining for antibodies developed against the normal glycoform of the antigen; in this case, the broad epithelial expression of normally-glycosylated MUC1 would credential it as an unsafe target for CAR T cells. Similar arguments could be made for the lack of cancer-specific splice variants in public IHC databases, such as EGFRvIII vs. EGFR expression. In addition, false positives and false negatives are problems not yet resolved and the sensitivity of IHC for low-expressing antigens may not be sufficient to select CAR targets for solid tumors. These limitations and pitfalls are well discussed in a previous review (59). New technologies, such as single cell RNA sequencing, may provide more accurate expression profiles that enable researchers to better predict efficacy and toxicity of novel CAR T cells.

## Strategies to expand therapeutic window of CAR T cell therapies

**Figure 2:**
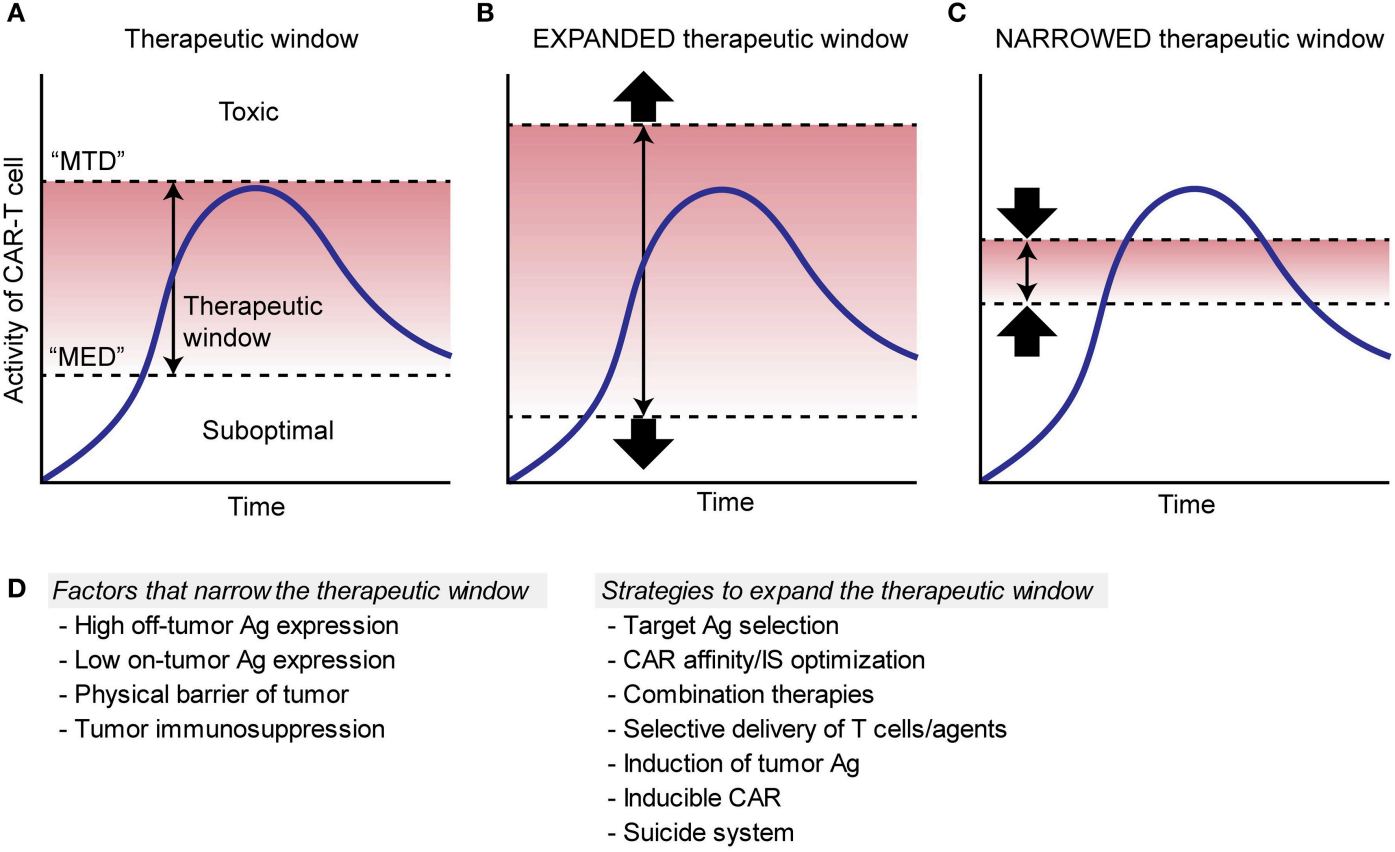
Schema to illustrate therapeutic window of CAR T cell therapies. **(A)**. Therapeutic window (red area) is determined as a range between the minimum effective dose (“MED”) and the maximum tolerated dose (“MTD”), where the therapy can achieve the highest therapeutic benefit without resulting in unacceptable toxicity. Blue lines show the kinetics of CAR T cells. **(B)**. Red area shows an expanded therapeutic window with decreased “MED” and/or increased “MTD,” which increase a chance for the CAR T cells to induce sufficient efficacy without toxicity. **(C)**. Red area shows a narrowed therapeutic window with increased “MED” and/or decreased “MTD,” which can cause toxicity and/or suboptimal efficacy. **(D)**. Factors that can narrow the therapeutic window and possible strategies to expand the window are listed.

“Therapeutic window” is a term originally from pharmaceutical toxicology and defined as a range of doses between efficacy and toxicity, achieving the highest therapeutic benefit without resulting in unacceptable toxicity; it is the range between the minimum effective dose (MED) and the maximum tolerated dose (MTD) (Figure 2A). Although the pharmacokinetics of engineered replicating cells are largely different from that of drugs, applying the concept of the therapeutic window to the field of ACTs will be valuable for optimizing the therapies. In CAR T cell therapies, targeting antigens expressed exclusively on tumor cells or antigens that are expressed only on non-critical tissues widens the therapeutic window as direct toxicity on vital tissues would not occur (Figure 2B). On the other hand, targeting antigens that are expressed in critical normal tissues/cells narrows the window by decreasing MTD (Figure 2C).

Determination of the therapeutic window cannot be resolved solely based on the profile of antigen expression. For instance, even in the case of a large differential in antigen expression by tumors and normal tissues, where the antigen is expressed at higher density in tumors, tumors may still be more resistant to CAR T cells than normal tissue due to inherent immunosuppression within the TME that does not exist within normal tissue. In this case, inhibition of T cell infiltration or induction of T cell hypofunction by the tumor would narrow the therapeutic window of CAR T cells by increasing the MED.

Given that truly tumor-specific target surface antigens have as of yet been rarely found, TAAs with shared expression of normal organs may be our only reasonable targets for the foreseeable future; therefore, strategies to expand the therapeutic window of CAR T cell therapy are necessary for the treatment of solid tumors. The possible approaches to expand therapeutic window include: (1) optimizing CAR affinity and sensing, (2) optimizing immunological synapse formation, (3) combination therapies, (4) local delivery of CAR T cells and therapeutic agents, (5) induction of target antigen expression, or (6) other modifications (Figure 2D).

## Optimizing CAR density, affinity and sensing

Although increasing CAR affinity enables recognition of antigens independent of target density (60), that action may cause serious adverse effects, namely on-target, off-tumor toxicity, and reduce the capability of sequential target tumor killing. Therefore, it is important to address rational strategies to determine ideal CAR affinity. The construction of affinity-tuned scFvs using light-chain exchange technology is one of the most feasible methods to measure the optimal affinities of CARs. With this method, Drent et al. identified that CD38-CAR T cells with ~1000-fold lower affinity to the original antibody that exhibited optimal proliferation, cytokine production, and cytotoxicity *in vitro* and *in vivo*, but spared normal tissue, compared with high-affinity CAR T cells (61).

Increasing the affinity of scFvs beyond a defined threshold (K_d_ <10^−8^ M) does not necessarily induce improved T cell activation (37, 62, 63). For instance, μM affinity CAR T cells exhibited superior cytokine production, expansion and antitumor efficacy, and less systemic off-tumor toxicity compared to nM affinity CAR T cells (64). These reports suggest that CAR T cells with affinities above a defined threshold are not necessarily required, or rather it may be important to generate CARs with varied affinities for the same epitope, and to identify the lowest affinity at which those epitope-specific CAR T cells can exhibit maximal cytolytic, proliferative, and safety potential.

Apart from changing scFv affinity, regulating the level of surface CAR expression is an important factor to induce ideal CAR signaling. CAR T cell function is governed by CAR density as well as target antigen density, where low expression of either can result in limited functionality and sensitivity of CAR T cells (36). On the other hand, continuous signaling (tonic signaling) through CAR can occur depending on the CAR structure and high CAR density, which can induce inferior antitumor effects and T cell engraftment *in vivo* by increasing T cell differentiation, exhaustion and activation induced cell death (AICD) (65, 66). Modifying CAR density while maintaining expression under the threshold required for tonic signaling induction will be required to induce sufficient anti-tumor efficacy and to keep safety potential for each target antigen and CAR construct. Another approach is combinatorial antigen recognition through two different antigens on tumor cells. Split-signaling CAR T cells have been engineered such that CAR-1 drives only the activation signal (signal 1) of CD3ζ and CAR-2 drives only co-stimulation (signal 2) through co-stimulatory molecules, such as CD28 and 4-1BB. Thereby, CAR T cells can be optimally activated only upon recognition of two separate required antigens simultaneously (“AND” logic gated CAR). This approach has been tested in CAR T cells with split signals utilizing anti-HER2 and anti-MUC1 CARs (67) or anti-CD19 and anti-PSMA CARs (68) in pre-clinical models. Conversely, CAR T cells can be modified so that CARs can drive full signaling upon recognizing either of two different antigens by expressing two CARs or a single CAR with tandem antigen binding domains (“OR” logic gated CAR) (69–71). Engineering T cells with “AND” logic CARs enables more specific and safer targeting and those with “OR” logic CARs potentially overcomes low target antigen expression and tumor escape by target antigen loss.

The use of adapter molecules to develop a “universal” CAR can be another attractive platform to overcome tumor heterogeneity in antigen expression and to make CAR T cell activities more conditional. Urbanska et al. described a biotin-binding immune receptor composed of an extracellular-modified avidin linked to an intracellular T cell signaling domain which can recognize tumor cells pre-treated with antigen-specific molecules such as mAb, scFv, or other tumor-specific ligands (72). Tamada et al. similarly described anti-FITC CAR which can target those pre-treated with FITC conjugated mAbs, and they demonstrated that anti-FITC CAR T cell activity can be attenuated by injecting FITC-labeled non-specific IgG Ab in a preclinical model (73). This platform enables flexible multiple tumor antigen targeting, which potentially prevents target antigen loss and ease off-tumor toxicity by dividing off-tumor toxicities. This system also enables control of CAR T cell activities by adjusting doses of adopter molecules, or more actively, by quenching CAR by adding the excess amount of non-specific tagged molecules. However, it is still unclear whether each adaptor will induce equal activity when ligated to the acceptor CAR molecule. For instance, it has been revealed that the length and composition of the CAR hinge influences the activity of the T cells and the epitope of the antibody also influences this (whether it is distal or proximal to the target cell membrane) (74–76). It will be needed to address these factors so that they each provide an optimal CAR signal. Another potential problem of this platform to translate to the clinic will be the immunogenicity of the adapter molecules.

## Optimizing immunological synapse formation

As discussed above, the CAR IS is more disorganized when compared to the well-organized bull's eye structure of TCR IS and is characterized by a multifocal pattern of Lck arrangement, decreased actin rings, and diffuse LFA-1 distribution. The remarkable capabilities of CAR IS are virtually instant induction of proximal signaling and rapid delivery of cytotoxic granules mediated by a faster migration of MTOC to the CAR IS compared to the TCR IS. These superiorities enable CAR T cells to dissociate quickly from destructed tumor cells and to mediate efficient serial killing.

Recently, several studies have reported important findings on how CAR design affects IS formation. Xiong et al. examined the quality of the CAR IS using CD19-specific CAR constructed with either CD28 or 4-1BB co-stimulatory domains and determined that CD28 plus 4-1BB-based third generation CARs are superior to CD28 based second generation CARs, as measured by IS structure, signaling and function (22). In comparison to bi-specific CAR T cells, CARs specific for two glioma-associated antigens, HER2 and IL13Rα2, exhibited significantly higher F-actin accumulation and increased polarization of the MTOC.

The structural characteristics of CAR IS are now in the beginning of elucidation. Although further studies are essential to reveal the correlation between CAR IS structure and the anti-tumor efficacy, modulation of CAR IS would be a great option of CAR T cell therapy if it is possible to increase the efficacy. There have been several attempts to improve the efficacy of CAR T cells by modifying CAR IS through immunomodulatory drugs (IMiDS), such as lenalidomide—a synthetic derivative of thalidomide. Lenalidomide improves CAR efficacy by increasing actin accumulation at the IS and is a promising combinatorial treatment for enhanced CAR activity (77, 78).

## Combination therapy

Combinatorial approaches may serve as a promising strategy to drive CAR T cell therapy toward solid tumors by overcoming tumor heterogeneity and expanding the therapeutic window. Oncolytic viruses (OVs) are promising agents for the treatment of solid tumors, and an oncolytic herpes virus expressing GM-CSF has been FDA-approved for the therapy of advanced melanoma based on therapeutic benefit in a clinical study (79). OVs can be programmed to specifically target, replicate in, and lyse cancer cells, while sparing normal cells. The release of virus progeny results in an exponential increase of the virus inoculum, which can cause direct tumor lysis while providing danger signals necessary to awaken the immune system (80). Furthermore, OVs can be genetically modified to express therapeutic transgenes selectively in the TME. Their ability to revert tumor immunosuppression while locally expressing therapeutic transgenes provides a rational strategy for combination with CAR T cell therapies. Indeed, we and other researchers reported enhanced CAR T cell efficacy by combining OVs expressing either cytokines (81), chemokines (82), an anti-PD-L1 minibody (83), a BiTE (84), or the combination of them against solid tumors in pre-clinical mouse models. We have shown that an oncolytic adenovirus expressing IL-2 and TNF-α enhanced the efficacy of mesothelin-redirected CAR T cells, which was associated with enhanced T cell infiltration to the tumor bed and reduced metastases (81). Murine TNF-α and murine IL-2 delivered by adenovirus could increase the efficacy of mesothelin-redirected CAR T cells in immunocompetent mice engrafted with highly immunosuppressive syngeneic LSL-Kras^G12D/+^;LSL-Trp53^R172H/+^;Pdx-1-Cre (KPC) mice derived-pancreatic ductal adenocarcinoma (PDA) tumor, whereas multiple injections of anti-mesothelin-CAR T cell monotherapy failed to suppress tumor growth. This combination approach enhanced the efficacy of CAR T cells and did not induce off-tumor toxicity.

Other combinatorial approaches include combination with agonistic antibodies specific for the 4-1BB costimulatory receptor (85), which can directly activate CAR T cells and also can reduce host immunosuppressive immune cells, such as Tregs or MDSCs.

## Local delivery of CAR T cells and therapeutic agents

Although efficient trafficking of CAR T cells to cerebrospinal fluid in patients with central nervous system (CNS) involved acute lymphoblastic leukemia has been reported (1), the response of primary or metastatic solid tumors in the CNS may be limited by the accessibility of CAR T cells. On the other hand, enhancing the strength of systemically administrated CAR T cells can raise safety concerns, as reported in HER2-redirected CAR T cell therapy. Direct administration of CAR T cells into the tumor bed is an optional route of drug delivery. Priceman et al. demonstrated that intraventricular delivery of HER2-CAR T cells shows antitumor activity against brain-metastatic breast cancer in orthotopic xenograft models, whereas intravenous delivery of HER2-CAR T cells achieved only partial antitumor responses in mice even at 10-fold higher doses compared with local or regional delivery to the brain (86). In confirmation of this administration route, intraventricular administration of IL13Rα2-targeting CAR T cells induced regression of all intracranial and spinal tumors in a patient with recurrent multifocal glioblastoma (87). We have tested intratumoral administration of mRNA-transduced anti-c-Met CAR T cells in patients with metastatic breast cancer (88) in a clinical trial and confirmed feasibility of this approach for clinical use.

Another approach is to engineer CAR-T cells to work only or dominantly in the tumor site. Han et al. developed the “masked CAR” system, which consists of a masking peptide that blocks the antigen-binding site and a protease-sensitive linker. The authors demonstrated that proteases commonly active in the TME (and presumably inactive in normal tissue) can cleave the linker and disengage the masking peptide, which enables CAR T cells to recognize target antigens only at the tumor site (89).

To overcome the immunosuppressive TME, local delivery of cytokines and chemokines, such as IL-18, IL-12, and the combination of CCL19 and IL-7 (90–92) or checkpoint blocking agents (93) by CAR T cells within the TME may help to overcome impediments to T cell infiltration and functionality. These approaches demonstrate enhanced therapeutic efficacy, while avoiding systemic adverse events in pre-clinical models.

## Induction of target antigen expression

As discussed here, target antigen density can govern the efficacy of CAR T cell therapy. In addition, the loss or down-regulation of target antigen is a major cause of tumor escape (94). Induction or re-induction of antigen expression on target cells may be an attractive approach to expand the therapeutic window. It has been reported that a sublethal dose of radiation can induce the expression of TAAs, such as mesothelin and CEA, on tumor cells (95). Epigenetic control may also modulate target antigen expression; in that vein, an anti-methylation drug, azacytidine (5-AZA), can re-induce CD20 expression on lymphoma cells after treatment, including after treatment with CD20-targeting mAb rituximab (96).

## Other modifications

Equipping CAR T cells with a suicide system, such as inducible caspase-9 (iCas9) (97, 98) or co-expression of truncated EGFR (99), will enhance the safety of CAR T cells. These systems can induce depletion of CAR T cells by administrating agents that trigger cell-intrinsic apoptosis or cell-extrinsic antibody-mediated depletion of the therapeutic cells. Transfection of T cells with mRNA encoding CAR enables transient expression of CAR (100) and is a technology that is suitable for early phase clinical trials if new antigens are targeted and dose-limiting toxicity may be predicted. Another approach for remote-controlled safety is an inducible CAR system, including a TET-inducible system (101), which enables drug-inducible control of CAR expression.

Lastly, the synthetic Notch (synNotch) system (102, 103) is another attractive platform for diverse and flexible modification of CAR T cells. SynNotch receptors can allow the addition of custom response programs to T cells upon antigen recognition. For instance, synNotch can drive tailored cytokine secretion, biased T cell differentiation, or local delivery of therapeutic payloads, such as antibodies, upon the recognition of the antigen. In addition, synNotch can be utilized to develop sophisticated antigen recognition by CAR T cells based on the Boolean “AND” logic gating.

## Conclusions

The treatment of solid tumors by CAR T cells is complex and multifactorial with a narrower therapeutic window than the targeting of CD19 for the treatment of B cell leukemia and non-Hodgkin lymphoma. Despite a growing list of clinical studies, remarkable responses have been rarely achieved with the exception of a case in glioblastoma with intraventricular delivery of the IL13Rα2 CAR. In this setting, establishment of strategies to expand the therapeutic window is critical. As outlined here, there are several promising approaches to achieve this in the preclinical setting and some of them are currently under investigation in clinical trials. The results of these and future clinical trials will elucidate a more refined path forward for solid tumor treatments.

There remain a lot of unknowns on tumor biology, the TME and CAR T cell biology. Fortunately, powerful tools to address these questions, such as emerging technologies in bioinformatics, mass spectrometry proteomics, mass cytometry, and single cell RNA sequencing, will allow us to access highly multiplexed and precise information on tumors, components of TME and immune cells. Moreover, maturation of technologies in gene-editing, such as the CRISPR/Cas9 system or synthetic biology, such as the synNotch system, will enable flexible design and engineering of T cells. Combining these technologies may lead to breakthroughs for CAR T cell therapies for the treatment of solid tumors.

Finally, several cases with unexpected severe toxicities have been reported when new CAR T cell therapies were first administrated to patients. Unfortunately, current technologies do not allow us to predict all the toxicities in the clinical setting; thus, only clinical trials can currently reveal information on safety and efficacy profiles of adoptive immunotherapies. Continuous development and refinement of preclinical models that can predict toxicity as well as the careful and rational planning and implementation of clinical trials will be crucial for further development of CAR T cell therapies.

## Author contributions

KW and SK conceptualized, wrote, and edited the manuscript. ADP and CHJ provided feedback and edited the manuscript.

### Conflict of interest statement

ADP and CHJ report intellectual property related to chimeric antigen receptor T cells licensed to Novartis. CHJ is a founder and has equity in Tmunity Therapeutics and receives commercial research grants from Novartis. CHJ is a consultant/advisory board member for Tmunity, Immune Design, and Celldex Therapeutics. The remaining author declares that the research was conducted in the absence of any commercial or financial relationships that could be construed as a potential conflict of interest.
